# Breast Implants and Lymphoma: What Is the Risk for Your Patient?

**Published:** 2017-09-01

**Authors:** Pamela Hallquist Viale

Breast implants require a surgical procedure and placement of medical devices designed to give the appearance of a natural-looking female breast. This surgery may be implemented for cosmetic reasons to augment the breast or to provide breast tissue after mastectomy or damage to the breast. In cancer care, advanced practitioners often care for patients who have this type of surgery after successful treatment of their original breast cancer. These implants are usually saline-filled or silicone gel–filled and may vary in size, thickness, and texture of the implant wall, as well as shape and contour (US Food and Drug Administration [FDA], 2017).

Reconstructive surgery of the breast with placement of a breast implant can help patients with breast cancer achieve a realistic-looking breast, providing confidence and satisfaction. However, in 2011, an unusual report of cancer occurring in a patient who had breast implants prompted the FDA to report a possible association between these implants and the subsequent development of anaplastic large cell lymphoma, or ALCL (FDA, 2017). This disease is not breast cancer or cancer of the actual breast tissue; instead, it is a specific lymphoma.

## INCIDENCE OF DISEASE

At the time of the report, because the incidence was rare, the FDA continued to compile data on the occurrence of this type of cancer in women with implants. During the collection of these data, it appeared that breast implant–associated anaplastic large cell lymphoma (BIA-ALCL) occurred more frequently with the implantation of breast implants with textured surfaces vs. smooth-surfaced devices (FDA, 2017).

The incidence of lymphoma has been noted worldwide. In Australia, 53 cases were confirmed with 3 deaths, prompting the Australian Therapeutic Goods Administration (TGA) to estimate the risk of BIA-ALCL to be between 1 in 1,000 to 1 in 10,000, although a published epidemiologic study in the *Journal of the American Medical Association* estimates the risk is more likely 1 in 300,000 (TGA, 2017; de Jong et al., 2008). The TGA also reports that most cases of BIA-ALCL may be cured by the removal of the implant and the capsule surrounding the implant; however, a small number of cases have been aggressive and have led to death (TGA, 2017).

The American Society of Plastic Surgeons (ASPS) and The Plastic Surgery Foundation reported that the disease occurs on average 8 years after original implantation. The specific aspects of the lymphoma include a pure T-cell lymphoma, without an anaplastic lymphoma kinase gene translocation (ALK–), and positivity for CD30 on immunohistochemistry (ASPS and The Plastic Surgery Foundation, 2017). 

As of February 1, 2017, the ASPS and The Plastic Surgery Foundation estimated that of 10 to 11 million women with implants worldwide, 118 specific cases have been identified in the literature. MD Anderson Cancer Center reports 160 cases noted from 15 different countries (ASPS and The Plastic Surgery Foundation, 2017). Patients most frequently present with a malignant effusion associated with the capsule; any patient with a seroma occurring more than 1 year after original surgical implantation should be considered suspicious for disease (Clemens, Nava, Rocco, & Miranda, 2017).

## MANAGEMENT OF BIA-ALCL

A comprehensive algorithm of the assessment and management of this complication has been developed by the ASPS and The Plastic Surgery Foundation (2017). Initial steps include an ultrasound of the breast and lymph node areas, usually followed by magnetic resonance imaging (MRI). If an effusion is present, a fine-needle aspiration is performed, followed by cytology, histology flow cytometry, and CD30 immunohistochemistry of the effusion; if a mass is present, a biopsy with the same cytology assessment is needed, followed by an oncology consult.

Once the histology of BIA-ALCL is confirmed, the incidence should be reported to the FDA and the PROFILE Registry. The PROFILE Registry is committed to increase the scientific data on this disease and promote better understanding of the role of breast implants and the development of primary ALCL (The Plastic Surgery Foundation, 2017). The registry site is part of The Plastic Surgery Foundation and provides a comprehensive list of helpful publications, resources, and links to associated articles on this complication.

The patient is referred to an oncologist, who will facilitate a multidisciplinary team consultation and comprehensive lymphoma workup and staging with bone marrow biopsy and positron emission tomography–computed tomography (PET-CT) scan. Management of this complication usually requires removal of the implant and the associated capsule of tissue. If the patient is diagnosed with localized disease, total capsulectomy should be followed by close monitoring and surveillance ultrasounds (possibly with CT). If advanced disease is present, capsulectomy followed by adjuvant therapy may be considered, using CHOP3 (cyclophosphamide, doxorubicin, vincristine, and prednisone) or a clinical trial (ASPS and The Plastic Surgery Foundation, 2017; Miranda et al., 2014; National Comprehensive Cancer Network, 2017). 

The data suggest that BIA-ALCL is highly curable in most patients, although a small number will have aggressive disease requiring more therapy with cytotoxics (Clemens et al., 2017). Because the disease has been linked to implants with textured surfaces, it is not recommended to reimplant with those devices, although several patients have received replacements with smooth implants following lymphoma therapy, with close follow-up recommended (Clemens et al., 2017).

## IMPLICATIONS FOR ADVANCED PRACTITIONERS

The FDA made original recommendations regarding the risk of BIA-ALCL in 2011 following the first reports of the disease. Manufacturers of implants have added warnings to the package inserts for those devices in the United States and Canada (Clemens et al., 2017). Advanced practitioners may be caring for this particular group of patients and should be aware of the importance of risk disclosure and identification of initial symptoms of BIA-ALCL. If patients are considering breast implantation, risk disclosure should include making patients aware of this rare disease and informing them of the common presenting symptoms (breast mass or delayed presentation of seroma or effusion) and the importance of medical follow-up if these symptoms occur (Clemens et al., 2017).

If you have a patient diagnosed with this rare complication, the FDA and the PROFILE Registry should be informed. Continuation of data collection on BIA-ALCL will contribute to our understanding of breast implants and ALCL, improving our ability to optimally care for this patient population.
